# Application progress of plant-mediated RNAi in pest control

**DOI:** 10.3389/fbioe.2022.963026

**Published:** 2022-08-08

**Authors:** Xiang Li, Xiaoguang Liu, Wenhui Lu, Xinming Yin, Shiheng An

**Affiliations:** State Key Laboratory of Wheat and Maize Crop Science/Henan International Laboratory for Green Pest Control/College of Plant Protection, Henan Agricultural University, Zhengzhou, China

**Keywords:** RNAi-based biopesticides, dsRNA-expressing transgenic plants, nuclear transformation, plastid transformation, pest control

## Abstract

RNA interference (RNAi)-based biopesticides are novel biologic products, developed using RNAi principles. They are engineered to target genes of agricultural diseases, insects, and weeds, interfering with their target gene expression so as to hinder their growth and alleviate their damaging effects on crops. RNAi-based biopesticides are broadly classified into resistant plant-based plant-incorporated protectants (PIPs) and non-plant-incorporated protectants. PIP RNAi-based biopesticides are novel biopesticides that combine the advantages of RNAi and resistant transgenic crops. Such RNAi-based biopesticides are developed through nuclear or plastid transformation to breed resistant plants, i.e., dsRNA-expressing transgenic plants. The dsRNA of target genes is expressed in the plant cell, with pest and disease control being achieved through plant-target organism interactions. Here, we review the action mechanism and strategies of RNAi for pest management, the development of RNAi-based transgenic plant, and the current status and advantages of deploying these products for pest control, as well as the future research directions and problems in production and commercialization. Overall, this study aims to elucidate the current development status of RNAi-based biopesticides and provide guidelines for future research.

## 1 Introduction

RNA interference (RNAi) is a conserved RNA-mediated regulatory mechanism in eukaryotic organisms (animals, plants, insects, virous, and nematatodes etc.). RNAi effector targets genes with homologous sequence pairing and then effectively silences or suppresses their expression at the transcriptional or post-transcriptional level. RNAi was first identified in 1990 by Napoli et al. who transferred the *chalcone synthase* gene into petunia to produce darker purple-colored flowers. However, most of the flowers turned white or floral white instead of becoming darker. This phenomenon wherein an exogenous transferred gene, homologous to an endogenous gene, results in the suppression of their expression, is called co-suppression by researchers (Napoli et al., 1990). A similar phenomenon was observed in *Neurospora crassa* and was termed quelling by Romano and Macino (1992). Fire et al. (1998) confirmed that double-stranded RNA (dsRNA) can suppress the expression of target genes more effectively than single-stranded RNA (ssRNA) in *Caenorhabditis elegans*. They defined this post-transcriptional silencing of specific genes as RNAi.

Advancements over 2 decades have led to the maturation of RNAi technology in the pharmaceutical field. Especially in the field of agricultural pest control, RNAi-based biopesticides are engineered to target genes of agricultural diseases such as fungi and other pathogenic microorganisms, insects, and weeds, interfering with their target gene expression so as to hinder their growth and alleviate their damaging effects on crops. Therefore, RNAi technology can be used to develop novel biopesticides with high efficiency, simplicity, and specificity, expected to bring another scientific and technological revolution and facilitate sustainable agricultural development.

The huge potential of RNAi-based biopesticides has prompted research institutes and agrochemical companies to further invest in research for the translation of this technology into innovative products (Jalaluddin et al., 2019). Several large agrochemical and biotechnology companies, including Bayer (Monsanto), Corteva, and Syngenta, are using this technology for the development of novel products, and they have already attained several major patents for RNAi applications. RNAi-based biopesticides are broadly classified into two categories: 1) Resistant plant-based plant-incorporated protectants (PIPs) and 2) Non-plant-incorporated protectants (non-PIPs). Non-PIP RNAi-based biopesticides follow the traditional chemical biopesticide application method and are designed to exert a pest/disease control role by the means of foliar spray, root-irrigation, or seed dressing. There are still many issues need to be assessed and solved before large-scale field application of non-PIP, such as large-scale production, cost control, dsRNA degradation, RNAi efficiency, environmental risk assessment, and resistance evolution. No fully commercialized non-PIP RNAi-based biopesticide products have been released to the market thus far (Liu et al., 2019; Kunte et al., 2020). Notably, Bayer has announced that their insecticide ‘ledprona’ for *Leptinotarsa decemlineata* control will be submitted to the Environment Protection Authority (EPA) in 2022, which is expected to be the first EPA-registered non-PIP insecticide.

PIP RNAi-based biopesticides, on the other hand, are novel biopesticides that combine the advantages of RNAi and resistant transgenic crops. At present, several products (MON87411 in 2017, DP23211 in 2021, VT4PRO in 2022) have been approved for marketing, all of which are based on the expression of dsRNA in transgenic plants for pest control. Here, we review the action mechanism and strategies of RNAi for pest management, the development of RNAi-based transgenic plant, and the current status and advantages of deploying these products for pest control, as well as the future research directions and problems in production and commercialization. Overall, this study aims to elucidate the current development status of RNAi-based biopesticides and provide guidelines for future research.

## 2 RNAi strategies for pest management

### 2.1 RNAi core mechanism of action

Typically, dsRNAs or ssRNAs folded into a hairpin structure (hairpin RNA or hpRNA, a type of dsRNA) induce silencing of downstream RNA. According to the mechanism of RNAi, dsRNA entering into organism are cleaved intracellularly to 21–23-bp siRNAs by the nucleic acid endonuclease RNaseIII containing canonical Drosha and/or Dicer. Subsequently, siRNAs bind to the Argonaute protein (AGO) and multiple enzymes during the effector phase, to form an RNA-induced silencing complex (RISC), which subsequently targeted sequence-complementary mRNAs and cleave them by the carried endonuclease (Bartel, 2004; Carthew and Sontheimer, 2009), thereby inhibiting its translation activity to a protein and thus causing silencing of the underlying gene (Chen et al., 2010).

### 2.2 Application potential of RNAi in pest control

RNAi-based biopesticides are dsRNAs of insect target genes synthesized based on gene sequence complementarity, and whose activity is a result of the silencing effect of specific mRNAs in target organism. Genes essential to pest growth and development can be alone or in combination, and they can be as potential target genes in RNAi-based pesticide development, providing a significant advantage of RNAi-based pesticides over chemical ones. About 25%–35% of the insect genome harbours essential genes, which can be potential candidates as targets for RNAi (Dietzl et al., 2007). The very wide range of targets available for RNAi-based pesticides makes the upfront development cost relatively low. In the United States, the development of a conventional chemical pesticide takes at least 12 years from inception to market entry, costing an average of $280 million. Additionally, a conventional transgenic crop could be in development for an average of 13 years, with investment costs of $130-$140 million. In contrast, the development of RNAi-based biopesticides requires only a $3-7 million investment and can be completed within approximately 4 years (Marrone, 2014; Marrone, 2019). Therefore, RNAi-based pesticides have a good prospect and application in pest control.

In the agricultural field, using RNAi technology to silence the expression of crucial genes can inhibit the development of insects and even kill them, thereby achieving pest control. In 2002, Bettencourt et al. were the first to demonstrate the application feasibility of RNAi technology in pest control with the evaluation of *Hemolin*-RNAi in *Hyalophora cecropia* (Bettencourt et al., 2002). In subsequent years, researchers have showed that exogenous synthetic dsRNA can induce RNAi effects in a variety of insects by feeding, injection, or spray methods (Kim et al., 2015; Vogel et al., 2019). In 2007, Baum et al. (2007) and Mao et al. (2007). confirmed in Coleoptera and Lepidoptera insects, respectively, that dsRNA expressed by transgenic plants could be transported into insects by feeding and induced a strong RNAi response, confirming the feasibility of PIP method for pest control.

## 3 Generation of dsRNA-expressing transgenic plants

The technical principle of the PIP method is to design dsRNA based on certain gene sequences of target pests and construct efficient dsRNA expression vectors to generate transgenic plant varieties, so as to enhance crop resistance and eventually achieve chemical-free pest control. Plants contain three organelles that carry genetic information: Nucleus, chloroplast (plastid), and mitochondria. Current technological limitations make the nucleus and chloroplasts the only organelles that can be genetically transformed. Depending on the genome of the transformed plant organelle, transgenic plants expressing insect-resistant dsRNAs can be divided into two categories: 1) nuclear transformation (NT) transgenic plants and 2) plastid transformation (PT) transplastomic plants.

### 3.1 Nuclear transformation-mediated RNAi-based insecticidal plants

#### 3.1.1 NT approach

Plant (cell) NT is a method of introducing vectors containing exogenous DNA into the cell’s nuclear genome by biological or physical means such as *Agrobacterium* transfection, particle bombardment, and protoplast transformation. It is the most widely used genetic engineering method thus far due to its higher success rate and easier process than PT method. Initially, RNAi-based insecticidal plants were mainly used in this approach and operated as follows: The target gene fragment is inserted into the modified T-DNA region and then transferred to the genome of the recipient plant through the modified vector using *Agrobacterium* transfection. Once stably incorporated into the nuclear genome it is transcribed into dsRNA or hpRNA. The vector used to express dsRNA usually contains a sequence of DNA spaced by an intervening fragment with reverse complementarity. The sequence in the vector was transcribed into RNA in the cell and then folded in pairs to form a dsRNA with a loop structure to induce RNAi effect (Wesley et al., 2001). This method demonstrates significant effectiveness for the management of serious agricultural pests such as *Helicoverpa armigera*, *L. decemlineata*, *Nilaparvata lugens*, and aphids (Table 1).

**TABLE 1:** Nuclear transformation-mediated dsRNA-expressing transgenic plants in pest control.

Transgenic plants	Insects	Target genes	References
*Nicotiana tabacum*	*Helicoverpa armigera*	*CYP6AE14*	Mao et al. (2007)
		*ecdysone receptor*	Zhu et al. (2012)
		*hormone receptor 3*	Xiong et al. (2013)
		*glutathione S-transferase 16*	Shabab et al. (2014)
		*Chitinase*	Mamta et al. (2016)
		*acetylcholinesterase 1*	Saini et al. (2018)
	*Myzus persicae*	*Rack1*	Pitino et al. (2011)
		*MpC002*	
		*hunchback*	Mao and Zeng (2014)
		*CYP82E4v1*	Zhao et al. (2016)
		*cathepsin L*	Rauf et al. (2019)
	*Bemisia tabaci*	*v-ATPaseA*	Thakur et al. (2014)
		*acetylcholinesterase*	Malik et al. (2016)
		*ecdysone receptor*	
		*aquaporin*	Raza et al. (2016)
		*α-glucosidase*	
	*Manduca sexta*	*CYP6B46*	Kumar et al. (2012)
	*Spodoptera litura*	*chitin synthase A*	Rana et al. (2020)
	*Chilo partellus*		
	*Plutella xylostella*		
	*Maruca vitrata*		
*Arabidopsis thaliana*	*Helicoverpa armigera*	*arginine kinase*	Liu et al. (2015)
	*Myzus persicae*	*serine protease*	Bhatia et al. (2012)
		*cuticular protein*	Bhatia and Bhattacharya (2018)
	*Bemisia tabaci*	*glutathione S-transferase s5*	Eakteiman et al. (2018)
*Gossypium hirsutum*	*Helicoverpa armigera*	*CYP6AE14*	Mao et al. (2011)
		*HMG-CoA reductase*	Tian et al. (2015)
		*hormone receptor 3*	Han et al. (2017)
*Oryza sativa*	*Nilaparvata lugens*	*hexose transporter*	Zha et al. (2011)
		*Carboxypeptidase*	
		*trypsin-like serine protease*	
		*ecdysone receptor-c*	Yu et al. (2014)
		*glutathione S-transferase*	Yang et al. (2020)
*Triticum aesticum*	*Sitobion avenae*	*Carboxylesterase*	Xu et al. (2014)
		*Chitinase*	Zhao et al. (2018)
		*Gqα*	Hou et al. (2019)
		*zinc finger protein*	Sun et al. (2019)
*Glycine max*	*Tetranychus urticae*	*coatomer subunit α*	Dubey et al. (2017)
		*aquaporin 9*	
	*Leguminivora glycinivorella*	*ribosomal protein P0*	Meng et al. (2017)
*Zea mays*	*Diabrotica virgifera virgifera*	*v-ATPase A*	Baum et al. (2007)
		*vitellogenin receptor*	Niu et al. (2017)
		*boule*	
*Solanum tuberosum*	*Diabrotica virgifera virgifera*	*ecdysone receptor*	Hussain et al. (2019)
	*Leptinotarsa decemlineata*	*cuticular protein*	Naqqash et al. (2020)
		*glutathione synthetase*	
		*cytochrome P450 monoxygenases*	
*Lactuca sativa*	*Bemisia tabaci*	*v-ATPase A*	Ibrahim et al. (2017)
*Brassica juncea*	*Lipaphis erysimi*	*sucrase 1*	Dhatwalia et al. (2022)
*Sugarcane (Saccharum spp.)*	*Sphenophorus levis*	*V-ATPase E*	Mohan et al. (2021)

#### 3.1.2 Applications of NT-mediated dsRNA-expressing transgenic plants in pest control

##### 3.1.2.1 Insect pests with chewing mouthparts

*Diabrotica virgifera virgifera* was one of the first and most successful insects to be controlled using the NT-mediated PIPs technique. *D. v. virgifera* is a highly damaging agricultural pest that causes up to $1 billion in economic losses annually in the United States. Most importantly this pest has developed resistance to chemical insecticides, transgenic Bt crops, and even crop rotation practices (Levine et al., 2002; Gray et al., 2009; Wangila et al., 2015). However, this pest is very sensitive to RNAi-based approaches. Genetically modified maize expressing dsRNA of the *V-ATPase A* gene of *D. v. virgifera* can suppress the expression of the target gene in the pest’s intestinal cells, resulting in 50% less damages in the genetically modified variety compared with the respective wild-type (Baum et al., 2007). Since then, several studies have demonstrated the feasibility of applying RNAi-based approaches for the control of *D. v. virgifera* (Zhang et al., 2013; Fishilevich et al., 2016; Fishilevich et al., 2019).

Widespread cultivation of transgenic Bt maize leads to gradual development of resistance in *D. v. virgifera*. The researchers found that feeding with ds*Snf7* exhibited superior control of *D. v. virgifera* larvae. Subsequently, transgenic Bt maize xpressing ds*Snf7* have been developed. As a result, this approach was combined with the insecticidal protein which has established high effectiveness, and their synergistism increased the control effect and slowed the evolution of pest resistance to Bt (Baum et al., 2007; Bolognesi et al., 2012). Thus RNAi-based resistant crop breeding strategy is effective for *D. v. virgifera* control, laying a solid foundation for commercial application of this technology. Based on the above research, Bayer (Monsanto) company was subsequently approved to market the first transgenic maize (trade name: MON87411) expressing both dsRNA and Bt protein for *D. v. virgifera* control in 2017.

PIP approach can also be applied to control lepidopteran insects. A pioneering study by Mao et al., in 2007 demonstrated improved defense in transgenic tobacco against *H. armigera* larvae by expressing exogenous dsRNA. The resistant tobacco plant was developed by deploying the *CYP6AE14* gene, which plays a crucial role in the metabolism of plant secondary metabolites, such as gossypol, by *H. armigera*. After *H. armigera* larvae fed on transgenic tobacco expressing a hpRNA targeting *CYP6AE14*, its expression level was significantly downregulated, resulting in reduced tolerance of *H. armigera* to gossypol. When *H. armigera* larvae were then fed with artificial feed containing gossypol, their growth and development were significantly inhibited (Mao et al., 2007). A follow-up study suggested that the simultaneous additional transfer of the *Cysteine protease* gene into cotton could further augment the insect-resistant effect of RNAi (Mao et al., 2013). Other target genes such as *EcR*, *HR3*, *GST16*, *AK*, and *HMGR* have also been exploited to generate RNAi-based transgenic crops for cotton bollworm control, with encouraging results (Zhu et al., 2012; Xiong et al., 2013; Shabab et al., 2014; Liu et al., 2015; Tian et al., 2015; Han et al., 2017).

Transgenic plants expressing dsRNA in combination with Bt have also been used in the control of *H. armigera*. A Bt insecticidal protein was combined with RNAi to generate cotton expressing a Bt toxin along with dsRNA that inhibits juvenile hormone synthesis and transport-related genes in *H. armigera*. This method not only improved the *H. armigera* control effect on transgenic cotton but also delayed *H. armigera* resistance build-up to Bt (Ni et al., 2017). However, since the RNAi efficiency in lepidopteran insects is far less than that of *D. v. virgifera*, no commercially available product has yet been developed.

##### 3.1.2.2 Insect pests with piercing-sucking mouthparts

RNAi-based insect-resistant strategy is also effectively used for insect pests with piercing-sucking mouthparts. Specifically, the expression of target genes in *N. lugens* was significantly suppressed, after feeding on transgenic rice expressing the respective dsRNA (Zha et al., 2011). Feeding *Myzus persicae* with plant-expressed dsRNAs for *MpC002* in the salivary gland and *Rack1* in the intestinal resulted in the suppression of two-thirds of target gene expression and a significant reduction in their progeny population (Pitino et al., 2011). Expression of the *hunchback* gene dsRNA in transgenic tobacco similarly enhanced resistance to *M. persicae* (Mao and Zeng, 2014). Sun et al. (2019) conducted a more detailed study using *Sitobion avenae*. They added a variety of dsRNA synthesized *in vitro* to artificial diet, and screened out a target gene *SaZFP*, whose suppression results in high lethality. Subsequently, a dsRNA segment of this gene was expressed in wheat. The mortality of *S. avenae* was increased significantly after 6 days of feeding on the transgenic wheat, eventually reaching an 80% aphid mortality after 18 days.

With the current progress in biotechnology tools, constructing transgenic crops expressing dsRNA using NT method has become a common and effective strategy for insect resistance. However, NT method possesses certain limitations, such as targeting a large and complex nuclear genome. Additionally, the random integration of exogenous fragments into the genome causes difficulty in controlling *Agrobacterium* transfection. NT also frequently results in low expression levels of exogenous genes and genetic instability (Pedras et al., 2011; Kaplanoglu et al., 2022). Additionally, most of the dsRNAs expressed by NT method were cleaved into siRNAs in the plant cells due to the action of endogenous Dicer enzymes in the plant cytoplasm (Xie et al., 2004). Therefore, both long-stranded dsRNA and processed siRNA are present in NT plants, lowering the insect control effectiveness. PT approaches can provide a solution to this, as Dicer is not present. Thus chloroplast-transformed plants express only long-stranded dsRNA, resulting in a significantly higher insect lethality compared to NT plants.

### 3.2 Plastid transformation-mediated RNAi-based insecticidal plants

#### 3.2.1 PT method

Plastid, a plant-specific organelle, is a general term used for various organelles such as proplastids, chloroplasts, leucoplasts, and amyloplasts. The plastid genome is relatively small, ranging in size from 120 to 200 kb. Plastid genes are arranged in clusters, and are co-expressed as a polycistron, with apparent prokaryotic features (Svab and Maliga, 1993). *Chlamydomonas reinhardtii* was the original model organism used to successfully express exogenous genes into chloroplasts by particle gun bombardment, thus demonstrating the feasibility of PT (Smith et al., 2011). Genetic engineering of the plastid genome emerged in the early 1990 s and was initially successful in tobacco and subsequently in tomato, potato, lettuce, and soybean. In contrast to the traditional NT method of plant cells (mediated by *Agrobacterium*, with random insertion of T-DNA into the nuclear genome of the plant), PT is achieved by homologous recombination and the targeted integration of exogenous genes into the plastid genome is carried out by particle bombardment. The use of *aadA* as a marker gene has led to a great improvement in PT efficiency (Kaplanoglu et al., 2022).

#### 3.2.2 Advantages of the PT method

PT has numerous advantages over the traditional NT technology: 1) High expression of exogenous genes. The plastid genome is present in multiple copies inside plant cells. A mature plant mesophyll cell contains 1,900–5,000 copies of the plastid genome, which can result in high levels of target gene expression; 2) Multi-gene co-transformation. The structure and expression pattern of plastid genes are similar to those of prokaryotes. Additionally, the small plastid genome can facilitate genetic manipulation to achieve multi-gene co-expression; 3) Higher environmental security. Most angiosperm plastids are inherited by the offspring through the maternal line. Since the pollen (male gametes) of the transformed plants does not contain transgenic components, plastid-engineered resistant crops cannot spread their genetic material with pollen, contributing to ecological stability; 4) Plastid gene expression without epigenetic silencing. The expression of plastid genes is free of epigenetic regulatory mechanisms such as methylation, acetylation, and histone modifications resulting in gene silencing. Therefore, exogenous genes can be stably and uniformly expressed in PT-mediated transgenic plants; 5) Regionalized gene expression products. Plastids have a relatively independent genetic system and both inner and outer membrane barriers. Therefore, the expression products of the target genes are confined within the plastids and have less effect on the physiological functions of other plant organelles(Bock, 2007; Yu et al., 2020).

#### 3.2.3 Application of PT-mediated dsRNA-expressing transgenic plants in pest control

In 2015, Zhang. et al. (2015a) first expressed the dsRNA of the lethal gene *β-actin* in potato chloroplasts for *L. decemlineata* control. dsRNA accumulation in leaves reached 0.4% of the total leaf RNA and was two to three orders of magnitude higher than that accumulated in NT controls. Additionally, no siRNA was detected in PT potatoes, confirming the absence of the RNAi pathway and the integrity of the expressed dsRNA in the plastids. Compared with wild-type and NT potato control plants, PT potatoes expressing the target dsRNA exhibited desirable pest-resistance levels. *L. decemlineata* larvae and adults stopped feeding on days 2 and 3, respectively, and all larvae died by day 4 following feeding, resulting in 100% insecticidal efficiency. Furthermore, the potato leaves were relatively intact. Thus, long-stranded dsRNA can be synthesized in large quantities in the chloroplasts to avoid being processed by Dicer in the plant cytoplasm. PT plants exhibited significant resistance to *L. decemlineata* and this finding paved the way for further applications of this approach to insect resistance. Since then, a series of studies proved that the construction of transgenic plants expressing dsRNA by PT method has a very remarkable effect on controlling pests with chewing mouthparts such as *H. armigera* (Jin et al., 2015), *Manduca sexta* (Poreddy et al., 2017), *Plutella xylostella* (Fu et al., 2020) and *Henosepilachna vigintioctopunctata* (Ren et al., 2021) (Table 2).

**TABLE 2:** Plastid transformation-mediated dsRNA-expressing transgenic plants in pest control.

Transgenic plants	Insects	Target genes	References
*Nicotiana tabacum*	*Leptinotarsa decemlineata*	*β-actin*	Zhang et al. (2015a)
		*Shrub*	
	*Helicoverpa armigera*	*CYP6AE14 v-ATPase A*	Jin et al. (2015)
		*chitin synthase*	
	*Manduca sexta*	*CYP6B46*	Poreddy et al. (2017)
	*Manduca quinquemaculata*		
	*Manduca sexta*	*v-ATPaseA*	Burke et al. (2019)
	*Bemisia tabaci*	*β-actin*	Dong et al. (2020)
	*Frankliniella occidentalis*	*β-actin*	Wu et al. (2022)
		*Tubulin v-TPase B*	
		*Snf7*	
*Nicotiana benthamiana*	*Helicoverpa armigera*	*acetylcholinesterase*	Bally et al. (2016)
*Solanum lycopersicum*	*Leptinotarsa decemlineata*	*v-ATPase*	Kaplanoglu et al. (2022)
	*Halyomorpha halys*		
	*Phenacoccus madeirensis*		
*Solanum tuberosum*	*Myzus persicae*	*β-actin*	Ren et al. (2021)
	*Henosepilachna vigintioctopunctata*		
	*Spodoptera litura*		
*Arabidopsis thaliana*	*Plutella xylostella*	*arginine kinase*	Fu et al. (2020)

The application of PT plants has target selectivity. Kaplanoglu et al. (2022) demonstrated that PT-mediated resistant tomato plant expressing dsRNA of the *v-ATPase A* gene exhibited significant feeding inhibition against leaf-chewing and lacerating- and flush-feeding insects, but poor feeding inhibition against sap-sucking insects. Wu et al. (2022) published a compromise study on the successful control for absorbing-mouthpart insects by PT-mediated RNAi plant. PT plants were evaluated for the control of *Frankliniella occidentalis*, which feeds with its rasping-sucking mouthparts. dsRNAs targeting four key genes of *F. occidentalis* (*β-actin*, *Tubulin*, *V-ATPase B*, and *Snf7*) were expressed through the plastid and nucleus, respectively. It was observed that *F. occidentalis* could ingest chloroplast contents (including dsRNA) during feeding on host plants. *F. occidentalis* could ingest more dsRNAs when feeding on PT plants than on NT plants. Subsequent bioassays revealed that PT plants resulted in higher *F. occidentalis* lethality. Additionally, PT plants were more robust due to significant reduction in the feeding of *F. occidentalis* on their leaves. Therefore, the PT-mediated RNAi strategy provides significantly more effective control of *F. occidentalis* than the traditional NT strategy (Wu et al., 2022).

Note that a major drawback when implementing this approach is the relatively small number of plant species that can be subjected to PT, approximately 20 species, including tobacco (Wu et al., 2017), tomato (Ruf et al., 2001), potato (Zhang J. et al., 2015), lettuce (Ruhlman, 2014), soybean (Dufourmantel et al., 2010), poplar (Wu et al., 2019), *Arabidopsis thaliana* (Ruf et al., 2019), and *Scoparia dulcis* (Kota et al., 2019), due to the limitations of PT technology. Therefore, it is crucial to further expand the range of crops that can efficiently undergo PT, especially focusing on economically important crops such as rice, wheat, and cotton.

## 4 Factors influencing insect resistance of RNAi-based transgenic plants

### 4.1 Target gene selection

The selection of target gene greatly affects the efficiency of plant-mediated RNAi. The target genes are usually the housekeeping genes of the target pest or are key genes in its growth and development. Silencing such genes has a greater probability to result in higher insect lethality, growth inhibition, or sterility. However, not all housekeeping genes are appropriate targets for RNAi, which may be attributed to the level of mRNA accumulation in insects. Whether the secondary structure of target gene mRNAs leads to higher susceptibility to exogenous dsRNA-induced silencing remains controversial. Consequently, Further studies are required to systematically elucidate the determinants of the sensitivity to target gene mRNA to RNAi.

### 4.2 dsRNA accumulation in plant tissues

dsRNA ingested by feeding transgenic plants enters cells via endocytosis and/or SID proteins (responsible for transporting dsRNA), and then induces the RNAi pathway and mRNA degradation of insect target genes. Unlike plants, insect lack RNA-dependent RNA polymerases, resulting in the inability to amplify the RNAi signal. Therefore, the RNAi effect is dependent on the amount of dsRNA entering insect cells. Consequently, the effect of RNAi on insect is positively correlated with the dose of dsRNA ingested, with a larger amount of dsRNA entering the cells leading to a higher mortality and/or growth impedance effect (Zhang et al., 2017). Therefore, dsRNA accumulation in plant tissues is a crucial factor that affects the efficiency of RNAi-based transgenic plants for insect control. Stronger promoters or transcription systems, and the use of PT to express dsRNA are promising solutions towards this goal (Zhang. et al., 2015b).

### 4.3 Internal environment of insects

The physiological state of the insect gut and hemolymph influences the effectiveness and efficiency of plant-mediated RNAi. Nucleases (dsRNases), that specifically degrade dsRNA were identified in the digestive fluid of *Bombyx mori.* They were secreted from the midgut cells of *B. mori* silkworms into the extracellular space and degraded in a non-sequence-dependent manner by ingested dsRNA (Arimatsu et al., 2007a; Arimatsu et al., 2007b). High activity dsRNases were localized in the intestines of many lepidopteran insect intestines, including *H*. a*rmigera*, *Chilo suppressalis*, and *Ostrinia furnacalis*, and rapidly degraded the dsRNAs ingested (Terenius et al., 2011; Singh et al., 2017; Zhang et al., 2017; Guan et al., 2018). These degradation enzymes prevent the the stability of long-stranded dsRNAs inside the insect’s gut, resulting in reduced RNAi effectiveness. Notably, the activities of dsRNases in coleopteran insects, such as *L. decemlineata*, *D. v. virgifera*, and *T. castaneum,* are weaker compared with those in lepidopteran pests (Singh et al., 2017). Therefore, coleopteran insects are more sensitive to RNAi-mediated control. At present, increasing the accumulation of dsRNA in transgenic plants may be the main approach to solve the insensitivity of lepidopteran insects to RNAi. Furthermore, the insect’s body biochemistry such as pH, ionic environment, and metabolite levels may affect the efficiency of plant-mediated RNAi.

## 5 Application and prospects of RNAi-based insecticidalplants

### 5.1 Development and application of RNAi-based insecticidalplants

On 15 June 2017, the United State Environmental Protection Agency approved MON87411, the first insect-resistant maize product expressing insect dsRNA in plants for the control of *D. v. virgifera*. This product carries the Bt protein (Cry3Bb1), the herbicide-resistant gene *cp4 epsps*, and the ds*snf7* against *D. v. virgifera*. It broadly belongs to the category of the PIP RNAi-based pesticides. Currently, it is licensed for cultivation in several countries, including the U.S., Canada, Brazil, and Japan. On 21 January 2021, Bayer announced, that the product has received a genetically modified organism (GMO) safety certificate (import and food/feed use) from the Ministry of Agriculture and Rural Affairs of China, further accelerating its commercialization. Bayer expects the product to be commercially grown in the United States. in 2022 and rolled out in Canada in 2023 (covering approximately 15 million acres over the next few years). Food Standards Australia New Zealand approved the RNAi-based herbicide-tolerant and insect-resistant maize product DP23211 for use in food-related crops on 9 February 2021. This transgenic maize carries ds*DvSSJ1* and *IPD072Aa* proteins to control corn rootworm control (*Diabrotica* spp.). On 2 March 2022, Bayer announced the commercial registrations from the EPA for the traits that will enable the commercialization of its newest corn product, VT4PRO™ with RNAi Technology, in the United States. Bayer plans to conduct large scale field testing of VT4PRO™ Technology during the 2022 and 2023 growing seasons with the potential to launch commercial volumes as early as 2024, pending state registrations.

### 5.2 Issues to be addressed during the large-scale application of RNAi-based insecticidal plants

#### 5.2.1 Resistance of pests to RNAi-based biopesticides

Pests and diseases will inevitably develop resistance to RNAi-based pesticides. Effectively addressing the biological resistance that may arise during long-term application will greatly extend the product’s service life. Thus, the potential underlying resistance mechanisms should be clarified to address and delay resistance development. Monsanto identified a population of *D. v. virgifera* resistant to ds*Snf7* in a laboratory setting through successive multi-generational screening. Further examination revealed that dsRNA obtained by insect feeding was not efficiently absorbed by the insect intestine in the resistant population. This population exhibited a general resistance to dsRNAs targeting other genes. Subsequent genetic analyses revealed mutations in recessive genes at the LG4 locus on the autosomal chromosomes of the resistant population. Nevertheless, crosses with sensitive strains resulted in the restoration of RNAi sensitivity (Khajuria et al., 2018). Such findings provide directions for addressing pest resistance in RNAi-resistant crops. Furthermore, the establishment of pest shelters and integrated pest management in combination with other pest-resistance management measures may extend the service life of dsRNA-based transgenic pest-resistant crops.

Genetic variation in pest populations is likely to result in single-nucleotide polymorphisms (SNPs) in target genes. The presence of SNPs that severely affect the complementarity of dsRNA with the target gene is likely to reduce RNAi efficiency and lead to resistance development by the pest, for that specific target (Scott et al., 2013; Yu et al., 2016). A bundant target resources are available for RNAi-based pesticides, and the generation of SNPs can be compensated by replacing target genes or sequences. In addition, changes in the internal environment of insects (such as increased RNase activity) are also important factors that mediate the development of insect resistance to RNAi biopesticides.

#### 5.2.2 Safety of dsRNA

The main component of RNAi-based biopesticides, dsRNA, is highly susceptible to degradation by various microorganisms, nucleases, or UV light in the environment. Numerous studies have confirmed that dsRNA is rarely accumulated in large quantities in soil (Blum et al., 1997; Levy-Booth et al., 2007; Dubelman et al., 2014). Therefore, RNAi-based biopesticides pose an extremely little environmental risk. Compared with non-PIP, the dsRNA expressed in transgenic plants is restricted to the plant tissue, further reducing the risk to the environment. Additionally, almost all biological cells can degrade nucleic acids into various bases and nucleosides for their metabolic needs.

Advances in sequencing technology and optimized bioinformatics platforms can address biosafety-related issues to some extent through multi-species sequence alignment. Multiple specialized databases such as FLIGHT, DRSC, and iBeetle-Base can provide RNAi phenotypes of key genes in *Drosophila* and beetles for target gene screening. Bioinformatics analysis tools (e.g., BLAST software) can be used to screen and identify RNAi targets by comparing genome-wide or transcriptome information of target and non-target organisms, thereby improving target gene screening specificity and sensitivity.

Similar to other chemical or biological pesticides, when RNAi-based pesticides are widely used as an agricultural product, they will enter the human body during ingestion as food. Therefore, the biosafety of RNAi-based pesticides in humans is a primary concern. The effects of RNAi and the range of target species are determined by dsRNA sequences. Since humans have a well-developed innate immune system, dsRNAs that do not match the human genome sequence will be regarded as exogenous invaders and recognized and degraded by multiple immune mechanisms (DeWitte-Orr et al., 2009; Whitehead et al., 2011). Furthermore, compared with the traditional transgenic plants expressing Bt insecticidal protein, the expression products of RNAi-based transgenic plants are nucleic acids rather than proteins, which have less impact on plant nutrient metabolism and reduce the potential risks to the environmental organisms.

High-quality genomic information enables the screening and selection of effective and specific RNAi target genes to avoid biosecurity and ecological security issues. However, many non-target organism species have not been fully sequenced, which poses a challenge for the development of RNAi-based pesticides, because they may bind to non-target genes in species for which no sequence information is available. Thus, conducting effective ecological safety assessments is difficult for species without genomic information (Zotti and Smagghe, 2015; Yin et al., 2016; Liu et al., 2019).

### 5.3 Prospects

The increasing global population and its growing living standards lead to higher demand for agricultural products demand higher in terms of quantity and quality. According to the UN forecast, the total global population will grow to 9.7 billion by 2050, and the food demand will increase by approximately 73%. Therefore, the continuous improvement of crop yield and quality without impairing the environment and harming the ecosystem is the focus of future agricultural research. RNAi-based pesticides have been shown to have many advantages such as a wide target range, low development cost, and high sustainability. Therefore, RNAi-based pesticides can satisfy important prerequisites such as quality and safety of agricultural products, ecological safety, and environmental security. Thus, they can serve as a new type of green biopesticide with great future development and application potential.

As a new class of biopesticides, relevant key scientific issues such as the mechanism of action of dsRNA, feasibility of dsRNA in pest control, screening of effective target genes, and the way to deliver dsRNA to target organisms have been largely assessed and solved. However, most of existing studies are limited to laboratory or small-scale experimental environments. As it is a novel biotechnological approach, many issues will be encountered during its large-scale application in the field. The maintenance of insect control efficacy and the management of biological resistance will ultimately determine the service life of the technology and its products. It should be emphasized that commercial dsRNA-expressing transgenic plant varieties often co-express Bt proteins, which may pose environmental risks and public resistance. In the future research, the cultivation of transgenic plant varieties that only express dsRNA should be strengthened. In addition, dsRNAs can be engineered for periodic or tissue-specific expression in resistant plants, depending on the behavioral characteristics of pests. MicroRNAs play an important regulatory role in the physiological and biochemical processes of insects, which also have potential as candidates to be the main components of RNAi biopesticides.

Furthermore, a major hurdle is that not all plants are amenable to genetic modification for the development of commercial transgenic varieties resistant to diseases and insects. Even if a particular crop is well suited for transgenesis, it may not be possible to achieve transgenic manipulation of the same crop for control of multiple pests and diseases. As such, non-PIP dsRNA formulations can be used synergistically in the development of RNAi-based novel pesticides for direct application to the surface of crops. The large-scale application of RNA biopesticides involves multidisciplinary knowledge, such as nanomaterials science, bioinformatics, and molecular biology. Additionally, the large-scale commercial deployment cost and the dynamic interactions between plants and pests need to be fully considered to effectively silence key gene expression and avoid adverse effects on non-target organisms. Furthermore, scholars should focus on the species-specific control properties of RNAi-based pesticides to accelerate the advancement of related technologies and their large-scale application in the field.

## References

[B1] Arimatsu Y. Furuno T. Sugimura Y. Togoh M. Ishihara R. Tokizane M. (2007a). Purification and properties of double-stranded RNA-degrading nuclease, dsRNase, from the digestive juice of the silkworm. Bombyx Mori J. Insect Biotechnol. Sericol. 76, 57–62. 10.11416/jibs.76.1_57

[B2] Arimatsu Y. Kotani E. Sugimura Y. Furusawa T. (2007b). Molecular characterization of a cDNA encoding extracellular dsRNase and its expression in the silkworm, *Bombyx mori* . Insect Biochem. Mol. Biol. 37 (2), 176–183. 10.1016/j.ibmb.2006.11.004 17244546

[B3] Bally J. McIntyre G. J. Doran R. L. Lee K. Perez A. Jung H. (2016). In-plant protection against *Helicoverpa armigera* by production of long hpRNA in chloroplasts. Front. Plant Sci. 7, 1453. 10.3389/fpls.2016.01453 27746796 PMC5040858

[B4] Bartel D. P. (2004). MicroRNAs: genomics, biogenesis, mechanism, and function. Cell 116 (2), 281–297. 10.1016/S0092-8674(04)00045-5 14744438

[B5] Baum J. A. Bogaert T. Clinton W. Heck G. R. Feldmann P. Ilagan O. (2007). Control of Coleopteran insect pests through RNA interference. Nat. Biotechnol. 25 (11), 1322–1326. 10.1038/nbt1359 17982443

[B6] Bettencourt R. Terenius O. Faye I. (2002). Hemolin gene silencing by ds-RNA injected into Cecropia pupae is lethal to next generation embryos. Insect Mol. Biol. 11 (3), 267–271. 10.1046/j.1365-2583.2002.00334.x 12000646

[B7] Bhatia V. Bhattacharya R. (2018). Host mediated RNAi of cuticular protein gene impaired fecundity in green peach aphid *Myzus persicae* . Pest Manag. Sci. 74, 2059–2068. 10.1002/ps.4900 29493869

[B8] Bhatia V. Bhattacharya R. Uniyal P. L. Singh R. Niranjan R. (2012). Host generated siRNAs attenuate expression of serine protease gene in *Myzus persicae* . PloS One 7 (10), e46343. 10.1371/journal.pone.0046343 23071558 PMC3468595

[B9] Blum S. A. E. Lorenz M. G. Wackernagel W. (1997). Mechanism of retarded DNA degradation and prokaryotic origin of DNases in nonsterile soils. Syst. Appl. Microbiol. 20 (4), 513–521. 10.1016/S0723-2020(97)80021-5

[B10] Bock R. (2007). Plastid biotechnology: prospects for herbicide and insect resistance, metabolic engineering and molecular farming. Curr. Opin. Biotechnol. 18 (2), 100–106. 10.1016/j.copbio.2006.12.001 17169550

[B11] Bolognesi R. Ramaseshadri P. Anderson J. Bachman P. Clinton W. Flannagan R. (2012). Characterizing the mechanism of action of double-stranded RNA activity against western corn rootworm (*Diabrotica virgifera virgifera* LeConte). PLoS One 7 (10), e47534. 10.1371/journal.pone.0047534 23071820 PMC3469495

[B12] Burke W. G. Kaplanoglu E. Kolotilin I. Menassa R. Donly C. (2019). RNA interference in the tobacco hornworm, *Manduca sexta*, using plastid-encoded long double-stranded RNA. Front. Plant Sci. 10, 313. 10.3389/fpls.2019.00313 30923533 PMC6426776

[B13] Carthew R. W. Sontheimer E. J. (2009). Origins and mechanisms of miRNAs and siRNAs. Cell 136 (4), 642–655. 10.1016/j.cell.2009.01.035 19239886 PMC2675692

[B14] Chen H. M. Chen L. T. Patel K. Li Y. H. Baulcombe D. C. Wu S. H. (2010). 22-nucleotide RNAs trigger secondary siRNA biogenesis in plants. Proc. Natl. Acad. Sci. U. S. A. 107 (34), 15269–15274. 10.1073/pnas.1001738107 20643946 PMC2930544

[B16] DeWitte-Orr S. J. Mehta D. R. Collins S. E. Suthar M. S. Jr M. G. Mossman K. (2009). Long double-stranded RNA induces an antiviral response independent of IFN regulatory factor 3, IFN-beta promoter stimulator 1, and IFN. J. Immunol. 183 (10), 6545–6553. 10.4049/jimmunol.0900867 19864603 PMC2885285

[B17] Dhatwalia D. Aminedi R. Kalia V. Pande V. Bhattacharya R. (2022). Host mediated attenuation of gut sucrase in mustard aphid *Lipaphis erysimi* impaired its parthenogenetic reproduction on Indian mustard Brassica juncea. Pest Manag. Sci. 78 (2), 803–811. 10.1002/ps.6694 34713547

[B18] Dietzl G. Chen D. Schnorrer F. Su K. C. Dickson B. J. Fellner M. (2007). A genome-wide transgenic RNAi library for conditional gene inactivation in *Drosophila* . Nature 448 (7150), 151–156. 10.1038/nature05954 17625558

[B19] Dong Y. Yang Y. Wang Z. Wu M. Fu J. Guo J. (2020). Inaccessibility to double-stranded RNAs in plastids restricts RNA interference in *Bemisia tabaci* (whitefly). Pest Manag. Sci. 76 (9), 3168–3176. 10.1002/ps.5871 32333833

[B20] Dubelman S. Fischer J. Zapata F. Huizinga K. Carson D. Uffman J. (2014). Environmental fate of double-stranded RNA in agricultural soils. PLoS One 9 (3), e93155. 10.1371/journal.pone.0093155 24676387 PMC3968063

[B21] Dubey V. K. Lee U. G. Kwon D. H. Lee S. H. (2017). Agroinfiltration-based expression of hairpin RNA in soybean plants for RNA interference against *Tetranychus urticae* . Pestic. Biochem. Physiol. 142, 53–58. 10.1016/j.pestbp.2017.01.004 29107247

[B22] Dufourmantel N. Dubald M. Matringe M. Canard H. Carcon F. Job C. (2010). Generation and characterization of soybean and marker-free tobacco plastid transformants over-expressing a bacterial 4-hydroxyphenylpyruvate dioxygenase which provides strong herbicide tolerance. Plant Biotechnol. J. 5 (1), 118–133. 10.1111/j.1467-7652.2006.00226.x 17207262

[B23] Eakteiman G. Koch R. M. Moshitzky P. Rincon N. M. Vassão D. G. Luck K. (2018). Targeting detoxification genes by phloem-mediated RNAi: a new approach for controlling phloem-feeding insect pests. Insect Biochem. Mol. Biol. 100, 10–21. 10.1016/j.ibmb.2018.05.008 29859812

[B24] Fire A. Xu S. Montgomery M. K. Kostas S. A. Driver S. E. Mello C. C. (1998). Potent and specific genetic interference by double-standed RNA in *Caenorhabditis elegans* . Nature 391 (6669), 806–811. 10.1038/35888 9486653

[B25] Fishilevich E. Vélez A. M. Storer N. P. Li H. Rangasamy M. Bowling A. J. (2016). RNAi as a management tool for the western corn rootworm, *Diabrotica virgifera virgifera* . Pest Manag. Sci. 72 (9), 1652–1663. 10.1002/ps.4324 27218412

[B26] Fishilevich E. Bowling A. J. Frey M. L. F. Wang P. H. Lo W. Rangasamy M. (2019). RNAi targeting of rootworm troponin I transcripts confers root protection in maize. Insect Biochem. Mol. Biol. 104, 20–29. 10.1016/j.ibmb.2018.09.006 30243801

[B27] Fu S. Liu Z. Chen J. Sun J. Z. Aun G. X. Jiang Y. X. (2020). Silencing arginine kinase/integrin β_1_ subunit by transgenic plant expressing dsRNA inhibits the development and survival of *Plutella xylostella* . Pest Manag. Sci. 76 (5), 1761–1771. 10.1002/ps.5701 31785188

[B28] Gray M. E. Sappington T. W. Miller N. J. Moeser J. Bohn M. O. (2009). Adaptation and invasiveness of western corn rootworm: intensifying research on a worsening pest. Annu. Rev. Entomol. 54, 303–321. 10.1146/annurev.ento.54.110807.090434 19067634

[B29] Guan R. B. Li H. Fan Y. J. Hu S. R. Christiaens O. Smagghe G. (2018). A nuclease specific to Lepidopteran insects suppresses RNAi. J. Biol. Chem. 293 (16), 6011–6021. 10.1074/jbc.RA117.001553 29500196 PMC5912458

[B30] Han Q. Wang Z. Z. He Y. X. Xiong Y. H. Lv S. Li S. P. (2017). Transgenic cotton plants expressing the *HaHR3* gene conferred enhanced resistance to *Helicoverpa armigera* and improved cotton yield. Int. J. Mol. Sci. 18 (9), E1874. 10.3390/ijms18091874 28867769 PMC5618523

[B31] Hou Q. L. Xu L. J. Liu G. Y. Pang X. M. Wang X. Zhang Y. F. (2019). Plant-mediated gene silencing of an essential olfactory-related *gqα* gene enhances resistance to grain aphid in common wheat in greenhouse and field. Pest Manag. Sci. 75 (6), 1718–1725. 10.1002/ps.5292 30525312

[B32] Hussain T. Aksoy E. Çalışkan M. E. Bakhsh A. (2019). Transgenic potato lines expressing hairpin RNAi construct of molting-associated *EcR* gene exhibit enhanced resistance against Colorado potato beetle (*Leptinotarsa decemlineata*, say). Transgenic Res. 28 (1), 151–164. 10.1007/s11248-018-0109-7 30607744

[B33] Ibrahim A. B. Monteiro T. R. Cabral G. B. Aragão F. J. L. (2017). RNAi-mediated resistance to whitefly (*Bemisia tabaci*) in genetically engineered lettuce (*Lactuca sativa*). Transgenic Res. 26 (5), 613–624. 10.1007/s11248-017-0035-0 28712067

[B34] Jalaluddin N. S. M. Othman R. Y. Harikrishna J. A. (2019). Global trends in research and commercialization of exogenous and endogenous RNAi technologies for crops. Crit. Rev. Biotechnol. 39 (1), 67–78. 10.1080/07388551.2018.1496064 30198341

[B35] Jin S. X. Singh N. D. Li L. B. Zhang X. L. Daniell H. (2015). Engineered chloroplast dsRNA silences cytochrome p450 monooxygenase, V-ATPase and chitin synthase genes in the insect gut and disrupts *Helicoverpa armigera* larval development and pupation. Plant Biotechnol. J. 13 (3), 435–446. 10.1111/pbi.12355 25782349 PMC4522700

[B36] Kaplanoglu E. Kolotilin I. Menassa R. Donly C. (2022). Plastid transformation of micro-tom tomato with a Hemipteran double-stranded RNA results in RNA interference in multiple insect species. Int. J. Mol. Sci. 23 (7), 3918. 10.3390/ijms23073918 35409279 PMC8999928

[B37] Khajuria C. Ivashuta S. Wiggins E. Flagel L. Moar W. Pleau M. (2018). Development and characterization of the first dsRNA-resistant insect population from western corn rootworm, *Diabrotica virgifera virgifera* LeConte. PLoS One 13 (5), e0197059. 10.1371/journal.pone.0197059 29758046 PMC5951553

[B38] Kim Y. H. Issa M. S. Cooper A. Zhu K. Y. (2015). RNA interference: applications and advances in insect toxicology and insect pest management. Pestic. Biochem. Physiol. 120, 109–117. 10.1016/j.pestbp.2015.01.002 25987228

[B39] Kota S. Hao Q. Narra M. Anumula V. Rao A. V. Hu Z. M. (2019). Improved plastid transformation efficiency in *Scoparia dulcis* L. J. Plant Biotechnol. 46 (4), 323–330. 10.5010/JPB.2019.46.4.323

[B104] Kumar P. Pandit S. S. Baldwin I. T. (2012). Tobacco rattle virus vector: A rapid and transient means of silencing *Manduca sexta* genes by plant mediated RNA interference. PloS One 7 (2), e31347. 10.1371/journal.pone.0031347 22312445 PMC3270032

[B40] Kunte N. McGraw E. Bell S. Held D. Avila L. A. (2020). Prospects, challenges and current status of RNAi through insect feeding. Pest Manag. Sci. 76 (1), 26–41. 10.1002/ps.5588 31419022

[B41] Levine E. Spencer J. L. Isard S. A. Onstad D. W. Gray M. E. (2002). Adaptation of the western corn rootworm to crop rotation: evolution of a new strain in response to a management practice. Am. Entomol. 48 (2), 94–107. 10.1093/ae/48.2.94

[B42] Levy-Booth D. J. Campbell R. G. Gulden R. H. Hart M. M. Powell J. R. Klironomos J. N. (2007). Cycling of Extracellular DNA in the soil environment. Soil Biol. Biochem. 39 (12), 2977–2991. 10.1016/j.soilbio.2007.06.020

[B43] Liu F. Wang X. D. Zhao Y. Y. Li Y. J. Liu Y. C. Sun J. (2015). Silencing the *HaAK* gene by transgenic plant-mediated RNAi impairs larval growth of *Helicoverpa armigera* . Int. J. Biol. Sci. 11 (1), 67–74. 10.7150/ijbs.10468 25552931 PMC4278256

[B44] Liu S. Jaouannet M. Dempsey D. M. A. Imani J. Coustau C. Kogel K. H. (2019). RNA-based technologies for insect control in plant production. Biotechnol. Adv. 39, 107463. 10.1016/j.biotechadv.2019.107463 31678220

[B45] Malik H. J. Raza A. Amin I. Scheffler J. A. Scheffler B. E. Brown J. K. (2016). RNAi-mediated mortality of the whitefly through transgenic expression of double-stranded RNA homologous to acetylcholinesterase and ecdysone receptor in tobacco plants. Sci. Rep. 6, 38469. 10.1038/srep38469 27929123 PMC5143975

[B46] Mamta K. Reddy K. R. K. Rajam M. V. (2016). Targeting chitinase gene of *Helicoverpa armigera* by host-induced RNA interference confers insect resistance in tobacco and tomato. Plant Mol. Biol. 90 (3), 281–292. 10.1007/s11103-015-0414-y 26659592

[B47] Mao J. J. Zeng F. R. (2014). Plant-mediated RNAi of a gap gene-enhanced tobacco tolerance against the *Myzus persicae* . Transgenic Res. 23 (1), 145–152. 10.1007/s11248-013-9739-y 23949691

[B48] Mao Y. Cai W. J. Wang J. W. Hong G. J. Tao X. Y. Wang L. J. (2007). Silencing a cotton bollworm P450 monooxygenase gene by plant-mediated RNAi impairs larval tolerance of gossypol. Nat. Biotechnol. 25 (11), 1307–1313. 10.1038/nbt1352 17982444

[B49] Mao Y. B. Tao X. Y. Xue X. Y. Wang L. J. Chen X. Y. (2011). Cotton plants expressing *CYP6AE14* double-stranded RNA show enhanced resistance to bollworms. Transgenic Res. 20 (3), 665–673. 10.1007/s11248-010-9450-1 20953975 PMC3090577

[B50] Mao Y. B. Xue X. Y. Tao X. Y. Yang C. Q. Wang L. J. Chen X. Y. (2013). Cysteine protease enhances plant-mediated bollworm RNA interference. Plant Mol. Biol. 83 (1–2), 119–129. 10.1007/s11103-013-0030-7 23460027 PMC3755213

[B51] Marrone P. G. (2014). The market and potential for biopesticides. ACS Symp. Ser. 1172, 245–258. 10.1021/bk-2014-1172.ch016

[B52] Marrone P. G. (2019). Pesticidal natural products–status and future potential. Pest Manag. Sci. 75 (9), 2325–2340. 10.1002/ps.5433 30941861

[B53] Meng F. L. Li Y. Zang Z. Y. Li N. Ran R. X. Cao L. X. (2017). Expression of the double-stranded RNA of the soybean pod borer *Leguminivora glycinivorella* (Lepidoptera: Tortricidae) ribosomal protein *P0* gene enhances the resistance of transgenic soybean plants. Pest Manag. Sci. 73 (12), 2447–2455. 10.1002/ps.4637 28598538

[B54] Mohan C. Shibao P. Y. T. Paula F. F. P. Toyama D. Vieira M. A. S. Figueira A. (2021). hRNAi-mediated knock-down of *sphenophorus levis V-ATPase E* in transgenic sugarcane (saccharum spp interspecific hybrid) affects the insect growth and survival. Plant Cell Rep. 40 (3), 507–516. 10.1007/s00299-020-02646-5 33389048

[B55] Napoli C. Lemieux C. Jorgensen R. (1990). Introduction of a chimeric *chalcone synthase* gene into petunia results in reversible Co-suppression of homologous genes in trans. Plant Cell 2 (4), 279–289. 10.1105/tpc.2.4.279 12354959 PMC159885

[B56] Naqqash M. N. Gökçe A. Emre Aksoy E. Bakhsh A. (2020). Downregulation of imidacloprid resistant genes alters the biological parameters in Colorado potato beetle, *Leptinotarsa decemlineata* say (chrysomelidae: Coleoptera). Chemosphere 240, 124857. 10.1016/j.chemosphere.2019.124857 31726599

[B57] Ni M. Ma W. Wang X. F. Gao M. J. Dai Y. Wei X. L. (2017). Next-generation transgenic cotton: pyramiding RNAi and Bt counters insect resistance. Plant Biotechnol. J. 15 (9), 1204–1213. 10.1111/pbi.12709 28199783 PMC5552478

[B58] Niu X. P. Kassa A. Hu X. Robeson J. McMahon M. Richtman N. M. (2017). Control of western corn rootworm (*Diabrotica virgifera virgifera*) reproduction through plant-mediated RNA interference. Sci. Rep. 7 (1), 12591. 10.1038/s41598-017-12638-3 28974735 PMC5626700

[B59] Pedras M. S. Hossain S. Snitynsky R. B. (2011). Detoxification of cruciferous phytoalexins in botrytis cinerea: spontaneous dimerization of a camalexin metabolite. Phytochemistry 72 (2–3), 199–206. 10.1016/j.phytochem.2010.11.018 21176925

[B60] Pitino M. Coleman A. D. Maffei M. E. Ridout C. J. Hogenhout S. A. Wilson A. C. C. (2011). Silencing of aphid genes by dsRNA feeding from plants. PLoS One 6 (10), e25709. 10.1371/journal.pone.0025709 21998682 PMC3187792

[B61] Poreddy S. Li J. Baldwin I. T. (2017). Plant-mediated RNAi silences midgut-expressed genes in congeneric Lepidopteran insects in nature. BMC Plant Biol. 17 (1), 199. 10.1186/s12870-017-1149-5 29132300 PMC5683459

[B62] Rana S. Rajurkar A. B. Kumar K. K. Mohankumar S. (2020). Comparative analysis of chitin SynthaseA dsRNA mediated RNA interference for management of crop pests of different families of Lepidoptera. Front. Plant Sci. 11, 427. 10.3389/fpls.2020.00427 32362904 PMC7182115

[B63] Rauf I. Asif M. Amin I. Naqvi R. Z. Umar N. Mansoor S. (2019). Silencing cathepsin L expression reduces *Myzus persicae* protein content and the nutritional value as prey for *Coccinella septempunctata* . Insect Mol. Biol. 28 (6), 785–797. 10.1111/imb.12589 30980445

[B64] Raza A. Malik H. J. Shafiq M. Amin I. Scheffler J. A. Scheffler B. E. (2016). RNA interference based approach to down regulate osmoregulators of whitefly (*Bemisia tabaci*): Potential technology for the control of whitefly. PLoS One 11 (4), e0153883. 10.1371/journal.pone.0153883 27105353 PMC4841547

[B65] Ren B. L. Cao J. N. He Y. Q. Yang S. Zhang J. (2021). Assessment on effects of transplastomic potato plants expressing Colorado potato beetle *β-actin* double-stranded RNAs for three non-target pests. Pestic. Biochem. Physiol. 178, 104909. 10.1016/j.pestbp.2021.104909 34446185

[B66] Romano N. Macino G. (1992). Quelling: transient inactivation of gene expression in Neurospora crassa by transformation with homologous sequences. Mol. Microbiol. 6 (22), 3343–3353. 10.1111/j.1365-2958.1992.tb02202.x 1484489

[B67] Ruf S. Hermann M. Berger I. J. Carrer H. Bock R. (2001). Stable genetic transformation of tomato plastids and expression of a foreign protein in fruit. Nat. Biotechnol. 19 (9), 870–875. 10.1038/nbt0901-870 11533648

[B68] Ruf S. Forner J. Hasse C. Kroop X. Seeger S. Schollbach L. (2019). High-efficiency generation of fertile transplastomic *Arabidopsis* plants. Nat. Plants 5 (3), 282–289. 10.1038/s41477-019-0359-2 30778165 PMC6420123

[B69] Ruhlman T. A. (2014). Plastid transformation in lettuce (*Lactuca sativa* L.) by biolistic DNA delivery. Methods Mol. Biol. 1132, 331–343. 10.1007/978-1-62703-995-6_21 24599864

[B70] Saini R. P. Raman V. Dhandapani G. Malhotra E. V. Sreevathsa R. Kumar P. A. (2018). Silencing of *HaAce1* gene by host-delivered artificial MicroRNA disrupts growth and development of *Helicoverpa armigera* . PloS One 13 (3), e0194150. 10.1371/journal.pone.0194150 29547640 PMC5856398

[B71] Scott J. G. Michel K. Bartholomay L. C. Siegfried B. D. Hunter W. B. Smagghe G. (2013). Towards the elements of successful insect RNAi. J. Insect Physiol. 59 (12), 1212–1221. 10.1016/j.jinsphys.2013.08.014 24041495 PMC3870143

[B72] Shabab M. Khan S. A. Vogel H. Heckel D. G. Boland W. (2014). OPDA isomerase GST16 is involved in phytohormone detoxification and insect development. FEBS J. 281 (12), 2769–2783. 10.1111/febs.12819 24730650

[B73] Singh I. K. Singh S. Mogilicherla K. Shukla J. N. Palli S. R. (2017). Comparative analysis of double-stranded RNA degradation and processing in insects. Sci. Rep. 7 (1), 17059. 10.1038/s41598-017-17134-2 29213068 PMC5719073

[B74] Smith N. A. Eamens A. L. Wang M. B. (2011). Viral small interfering RNAs target host genes to mediate disease symptoms in plants. PLoS Pathog. 7 (5), e1002022. 10.1371/journal.ppat.1002022 21573142 PMC3088724

[B75] Sun Y. Sparks C. Jones H. Riley M. Francis F. Du W. (2019). Silencing an essential gene involved in infestation and digestion in grain aphid through plant–mediated RNA interference generates aphid-resistant wheat plants. Plant Biotechnol. J. 17 (5), 852–854. 10.1111/pbi.13067 30582665 PMC6471730

[B76] Svab Z. Maliga P. (1993). High-frequency plastid transformation in tobacco by selection for a chimeric *AadA* gene. Proc. Natl. Acad. Sci. U. S. A. 90 (3), 913–917. 10.1073/pnas.90.3.913 8381537 PMC45780

[B77] Terenius O. Papanicolaou A. Garbutt J. S. Eleftherianos L. Huvenne H. Kanginakudru S. (2011). RNA interference in Lepidoptera: an overview of successful and unsuccessful studies and implications for experimental design. J. Insect Physiol. 57 (2), 231–245. 10.1016/j.jinsphys.2010.11.006 21078327

[B78] Thakur N. Upadhyay S. K. Verma P. C. Chandrashekar K. Tuli R. Singh P. K. (2014). Enhanced whitefly resistance in transgenic tobacco plants expressing double stranded RNA of *V-ATPase A* gene. PloS One 9 (3), e87235. 10.1371/journal.pone.0087235 24595215 PMC3940430

[B79] Tian G. Cheng L. L. Qi X. W. Ge Z. H. Niu C. Y. Zhang X. L. (2015). Transgenic cotton plants expressing double-stranded RNAs target HMG-CoA reductase (HMGR) gene inhibits the growth, development and survival of cotton bollworms. Int. J. Biol. Sci. 11 (11), 1296–1305. 10.7150/ijbs.12463 26435695 PMC4582153

[B80] Vogel E. Santos D. Mingels L. Verdonckt T. W. Broeck J. V. (2019). RNA Interference in insects: protecting beneficials and controlling pests. Front. Physiol. 9, 1912. 10.3389/fphys.2018.01912 30687124 PMC6336832

[B81] Wangila D. S. Gassmann A. J. Petzold-Maxwell J. L. French B. W. Meinke L. J. (2015). Susceptibility of Nebraska western corn rootworm (Coleoptera: Chrysomelidae) populations to Bt corn events. J. Econ. Entomol. 108 (2), 742–751. 10.1093/jee/tou063 26470186

[B82] Wesley S. V. Helliwell C. A. Smith N. A. Wang M. B. Rouse D. T. Liu Q. (2001). Construct design for efficient, effective and high-throughput gene silencing in plants. Plant J. 27 (6), 581–590. 10.1046/j.1365-313x.2001.01105.x 11576441

[B83] Whitehead K. A. Dahlman J. E. Langer R. S. Anderson D. G. (2011). Silencing or stimulation? siRNA delivery and the immune system. Annu. Rev. Chem. Biomol. Eng. 2, 77–96. 10.1146/annurev-chembioeng-061010-114133 22432611

[B84] Wu Y. You L. L. Li S. C. Ma M. Wu M. T. Ma L. X. (2017). *In vivo* assembly in *Escherichia coli* of transformation vectors for plastid genome engineering. Front. Plant Sci. 8, 1454. 10.3389/fpls.2017.01454 28871270 PMC5566966

[B85] Wu Y. Y. Xu L. T. Chang L. Ma M. Q. You L. L. Jiang C. M. (2019). *Bacillus thuringiensis* Cry1C expression from the plastid genome of poplar leads to high mortality of leaf-eating caterpillars. Tree Physiol. 39 (9), 1525–1532. 10.1093/treephys/tpz073 31222266

[B86] Wu M. T. Dong Y. Zhang Q. Li S. C. Chang L. Loiacono F. V. (2022). Efficient control of western flower thrips by plastid-mediated RNA interference. Proc. Natl. Acad. Sci. U. S. A. 119 (15), e2120081119. 10.1073/pnas.2120081119 35380896 PMC9169809

[B87] Xie Z. X. Johansen L. K. Gustafson A. M. Kasschau K. D. Lellis A. D. Zilberman D. (2004). Genetic and functional diversification of small RNA pathways in plants. PLoS Biol. 2 (5), E104. 10.1371/journal.pbio.0020104 15024409 PMC350667

[B88] Xiong Y. H. Zeng H. M. Zhang Y. L. Xu D. Qiu D. W. (2013). Silencing the *HaHR3* gene by transgenic plant-mediated RNAi to disrupt *Helicoverpa armigera* development. Int. J. Biol. Sci. 9 (4), 370–381. 10.7150/ijbs.5929 23630449 PMC3638292

[B89] Xu L. J. Duan X. L. Lv Y. H. Zhang X. H. Nie Z. S. Xie C. J. (2014). Silencing of an aphid carboxylesterase gene by use of plant-mediated RNAi impairs *Sitobion avenae* tolerance of phoxim insecticides. Transgenic Res. 23 (2), 389–396. 10.1007/s11248-013-9765-9 24242160

[B90] Yang J. Sun X. Q. Zhu-Salzman K. Qin Q. M. Feng H. Q. Kong X. D. (2020). Host-induced gene silencing of Brown planthopper glutathione S-transferase gene enhances rice resistance to sap-sucking insect pests. J. Pest Sci. 94, 769–781. 10.1007/s10340-020-01296-6

[B91] Yin C. L. Shen G. Y. Guo D. H. Wang S. P. Ma X. Z. Xiao H. M. (2016). Insect base: A resource for insect genomes and transcriptomes. Nucleic Acids Res. 44 (D1), D801–D807. 10.1093/nar/gkv1204 26578584 PMC4702856

[B92] Yu R. Xu X. Liang Y. K. Tian H. G. Pan Z. Q. Jin S. H. (2014). The insect ecdysone receptor is a good potential target for RNAi-based pest control. Int. J. Biol. Sci. 10 (10), 1171–1180. 10.7150/ijbs.9598 25516715 PMC4261201

[B93] Yu X. D. Liu Z. C. Huang S. L. Chen Z. Q. Sun Y. W. Duan P. F. (2016). RNAi-mediated plant protection against aphids. Pest Manag. Sci. 72 (6), 1090–1098. 10.1002/ps.4258 26888776

[B94] Yu Y. H. Yu P. C. Chang W. J. Yu K. K. Lin C. S. (2020). Plastid transformation: how does it work? can it be applied to crops? what can it offer? Int. J. Mol. Sci. 21 (14), 4854. 10.3390/ijms21144854 PMC7402345 32659946

[B95] Zha W. J. Peng X. X. Chen R. Z. Du B. Zhu L. L. He G. C. (2011). Knockdown of midgut genes by dsRNA-transgenic plant-mediated RNA interference in the Hemipteran insect *Nilaparvata lugens* . PLoS One 6 (5), e20504. 10.1371/journal.pone.0020504 21655219 PMC3105074

[B96] Zhang H. Li H. Miao X. X. (2013). Feasibility, limitation and possible solutions of RNAi-based technology for insect pest control. Insect Sci. 20 (1), 15–30. 10.1111/j.1744-7917.2012.01513.x 23955822

[B97] Zhang J. Khan S. A. Hasse C. Ruf S. Heckel D. G. Bock R. (2015a). Full crop protection from an insect pest by expression of long double-stranded RNAs in plastids. Science 347 (6225), 991–994. 10.1126/science.1261680 25722411

[B98] Zhang H. Li H. C. Guan R. B. Miao X. X. (2015b). Lepidopteran insect species-specific, broad-spectrum, and systemic RNA interference by spraying dsRNA on larvae. Entomol. Exp. Appl. 155 (3), 218–228. 10.1111/eea.12300

[B99] Zhang J. Khan S. A. Heckel D. Bock R. (2017). Next-generation insect-resistant plants: RNAi-mediated crop protection. Trends Biotechnol. 35 (9), 871–882. 10.1016/j.tibtech.2017.04.009 28822479

[B100] Zhao D. Qin L. J. Zhao D. G. (2016). RNA interference of the nicotine demethylase gene CYP82E4v1 reduces nornicotine content and enhances *Myzus persicae* resistance in nicotiana tabacum L. Plant Physiol. biochem. 107, 214–221. 10.1016/j.plaphy.2016.04.016 27314515

[B101] Zhao X. Situ G. He K. Xiao H. Su C. Li F. (2018). Functional analysis of eight chitinase genes in rice stem borer and their potential application in pest control. Insect Mol. Biol. 27 (6), 835–846. 10.1111/imb.12525 30058753

[B102] Zhu J. Q. Liu S. M. Ma Y. Zhang J. Q. Qi H. S. Wei Z. J. (2012). Improvement of pest resistance in transgenic tobacco plants expressing dsRNA of an insect-associated gene *EcR* . PloS One 7 (6), e38572. 10.1371/journal.pone.0038572 22685585 PMC3369839

[B103] Zotti M. J. Smagghe G. (2015). RNAi technology for insect management and protection of beneficial insects from diseases: lessons, challenges and risk assessments. Neotrop. Entomol. 44 (3), 197–213. 10.1007/s13744-015-0291-8 26013264

